# Graphene Oxide Facilitates Transformation of Waste PET into MOF Nanorods in Ionic Liquids

**DOI:** 10.3390/polym15112479

**Published:** 2023-05-27

**Authors:** Deepa Gangaraju, Andikkadu Masilamani Shanmugharaj, Vadahanambi Sridhar

**Affiliations:** 1 Centre for Energy and Alternative Fuels, Department of Chemistry, VELS Institute of Science, Technology & Advanced Studies (VISTAS), Chennai 600117, Tamilnadu, India; deepa.vadahanambi@gmail.com; 2 Global Core Research Centre for Ships and Offshore Plants (GCRC-SOP), Pusan National University, Busan 46241, Republic of Korea

**Keywords:** ionic liquids, plastics recycling, metal organic framework, water splitting, graphene oxide

## Abstract

Although though ionic liquids (IL) are rapidly emerging as highly efficient reagents for the depolymerization of waste plastics, their high cost and adverse impact on the environment make the overall process not only expensive but also environmentally harmful. In this manuscript, we report that graphene oxide (GO) facilitates the transformation of waste polyethylene terephthalate (PET) to Ni-MOF (metal organic framework) nanorods anchored on reduced graphene oxide (Ni–MOF@rGO) through NMP (N-Methyl-2-pyrrolidone)-based coordination in ionic liquids. Morphological studies using scanning electron microscopy (SEM) and transmission electron microscopy (TEM) showed mesoporous three-dimensional structures of micrometer-long Ni-MOF nanorods anchored on reduced graphene substrates (Ni–MOF@rGO ), whereas structural studies using XRD and Raman spectra demonstrated the crystallinity of Ni-MOF nanorods. Chemical analysis of Ni–MOF@rGO carried out using X-ray photoelectron spectroscopy demonstrated that nickel moieties exist in an electroactive OH-Ni-OH state, which was further confirmed by nanoscale elemental maps recorded using energy-dispersive X-ray spectroscopy (EDS). The applicability of Ni–MOF@rGO as an electro-catalyst in a urea-enhanced water oxidation reaction (UOR) is reported. Furthermore, the ability of our newly developed NMP-based IL to grow MOF nanocubes on carbon nanotubes and MOF nano-islands on carbon fibers is also reported.

## 1. Introduction

Since the discovery of polyethylene terephthalate (PET) by J. R. Whinfield and J. T. Dickson of Calico Printers’ Association in 1941 [1], PET has become a ubiquitous material in our daily lives. In 2021, nearly 80.9 million tons of PET was produced, the majority of which was converted into ‘single use’ PET beverage containers or PET fibers. Recent estimates suggest a cumulative plastic waste generation of over 25,000 million metric tons by 2050, of which 36.4% is projected to be discarded in landfills or the environment. A similar fraction (36.4%) will be incinerated, and only 27.2% will be recycled [2]. Although PET is easy to recycle, according to PETRA (PET Resin Association), only 31% of waste PET is recycled in USA, 52% in Europe, and almost 90% in India [3]. The recycling of PET can be broadly divided into primary and secondary recycling techniques. Primary, mechanical, or physical recycling involves grinding, size reduction, or remolding of waste PET, which often results in low-value products with lower strength compared to virgin PET. These products usually end up as low-value products such as door mats, fibers etc. Another popular technique is either direct or indirect incineration of waste PET to produce energy, known as the waste-to-energy route. In the indirect route, waste PET, along with other plastic wastes, is converted to liquid fuels [4] that are eventually burnt to produce heat and electricity. Despite the ease of incineration technique, the process is unsustainable and results in the release of greenhouse gases that pollute the environment. Controlled pyrolysis can transform waste PET into carbon materials such as graphite [5], graphene [6], carbon nanotubes [7,8], etc. 

Secondary recycling generally involves the transformation of waste PET into monomers and short chain oligomers through chemolytic depolymerization. These monomers can be either repolymerized into PET (circular plastics economy) or short-chain oligomers, which can be used as building blocks for creating high-value chemicals (value-added plastic economy) [9]. Of these two options, value-added plastic economy has attracted increasing research efforts. Recent reports have shown that waste PET can be transformed into a wide variety of chemical building blocks such as Schiff bases [10], metal organic frameworks (MOF) [11], directly utilized in construction [12], etc. Among all the products derived from waste PET, the synthesis of MOF requires special mention. MOFs are inorganic–organic porous functional materials that have rapidly emerged as attractive materials for applications in immune therapy [13], gas separation and storage [14], water treatment [15], super capacitors [16], and barrier properties [17].

Generally, the process of depolymerizing waste PET requires four components: a solvent to dissolve PET, a transesterification catalyst (mostly metal), chain scission agents such as glycols, amines, alcohols, or alkalis, and finally, an external heat source, either conventional or microwave. Although nearly 100% of waste PET can be converted into monomers or oligomers, this technique suffers from drawbacks such as (i) difficulty to separate oligomers from the crude product, (ii) poor selectivity of the depolymerization process, which often results in a mixture of oligomers, and (iii) the requirement of environmentally harmful solvents such as chlorosolvents etc. One alternative is the use of ionic liquids. 

Ionic liquids (ILs) composed of organic cations and organic/inorganic anions are rapidly emerging as non-volatile and less flammable solvent media for a wide range of chemical reactions. By introducing structural and chemical functionalities on either or both cationic and anionic parts, it is possible to design “task specific” or tunable ILs with targeted properties and functions [18]. Moreover, the ability of ILs exist as either liquid or visco-elastic liquids over a wide range of temperatures and pressures have garnered significant attention in applications such as carbon dioxide sequestration [19], compatabilizers in polymer blends [20], anti-corrosion coatings [21], packaging [22], curing agents in polymers [23], polymer gel electrolytes [24], lignin fractionation [25], etc. 

Regarding depolymerization, ionic liquids are emerging as attractive materials for the depolymerization of lignin [26], polyamide [27], poly (ethylene terephthalate) (PET) [28], natural rubber [29], polycarbonates [30], cellulose [31], etc. Ionic liquids are particularly effective in depolymerizing heteroatom-containing polymers such as PET, nylon, cellulose, and lignin due to the ionic stepwise depolymerization process wherein bimolecular “nucleophilic substitution (SN2)” reactions occur at heteroatom sites with the cations of the ionic liquid. Since metals have a positive catalytic effect on depolymerization of waste PET, metal-based ionic liquids (MILs) can be more effective compared to all-organic ionic liquids. Metal-based ionic liquids (MILs) are a subclass of ionic liquids that exhibit the general characteristics of typical all-organic ILs and also possess additional functionalities such as magnetic and optical properties due to the presence of transition metals as cations. A wide range of metal-incorporated acidic ionic liquids based on quaternary ammonium [32], substituted pyridinium [33], and imidazolium cations [34,35] has been reported. However, all these traditional metal-incorporated acidic ionic liquids are not only expensive and involve complex multi-step synthesis procedures but are also highly toxic, which necessitates the development of safer, efficient, and inexpensive ionic liquids, especially for the depolymerization of waste PET. 

In this work, we report the conversion of waste PET into Ni-MOF nanorods using NMP-NiCl_2_ (N-methyl-2-pyrrolidone-nickel chloride) coordination ionic liquids. The rationale behind the choice of this ionic liquid system over the hundreds of ionic liquids available is that, when compared to the traditional non-biodegradable and potentially toxic imidazolium or pyridinium cation-based ionic liquids [36], NMP is not only biodegradable but is also known to be an excellent solvent for PET dissolution and its recovery [37]. Additionally, the ease of preparation at very low costs makes NMP-NiCl_2_ coordination ionic liquids a good alternative to imidazolium-based ionic liquids. This combination of ease of preparation and functionality has made NMP-NiCl_2_ ionic liquids useful in many applications such as drug delivery [38], desulfurization [39], extraction [40], catalysts in organic reactions [41], etc. Though a wide variety of metal ions such as Fe, Co, and Zn can be used for the preparation of NMP-based ionic liquids, the choice of nickel in this study was governed by our intention to use the synthesized Ni-MOF for urea-enhanced water splitting (UOR). 

However, irrespective of the size and shape of MOFs, the intrinsic low conductivity of MOFs makes it necessary to combine them with conductive carbon nanomaterials. Specifically, hybridizing Ni-MOFs with carbon nanotubes (CNT) [42] as well as graphene [43,44] has been reported as electro-catalysts, sensors [45], etc. However, in most cases, the synthesis of these hybrids involves a two-step procedure in which pre-synthesized Ni-MOFs are hybridized with chemically modified carbon nanotubes, graphene, or carbon black. This highlights the need for a simple one-pot technique for the synthesis of Ni-MOF on graphene. Therefore, in this manuscript, we aim to utilize the ability of NMP-NiCl_2_ coordination ionic liquid not only as the medium for the depolymerization of waste PET and its subsequent conversion into MOF nanorods, but also as a reducing agent to provide reduced graphene oxide. This reduced graphene oxide acts as the conductive substrate for anchoring the Ni-MOF nanorods to yield three-dimensional Ni–MOF@rGO structures. The utility of Ni–MOF@rGO in urea-enhanced water splitting was demonstrated.

## 2. Experimental

### 2.1. Materials and Methods

NMP (CAS number: 872-50-4, Reagent grade), nickel chloride (CAS number: 7718-54-9; 99% purity), graphite (CAS number: 7782-42-5; 99% purity) and urea (CAS number: 57-13-6; 99.5% purity) were purchased from Alfa-Aesar, Seoul, Republic of Korea, and used as received. Waste PET was obtained from discarded beverage bottles. NMP-based ionic liquids were prepared following the protocol reported by Li et al. [38]. Microwave irradiation was carried out using a domestic microwave oven manufactured by Daewoo, Seoul, Republic of Korea. The morphology of the nanostructures was examined using field-emission scanning electron microscopy (FE-SEM, Nova NanoSEM 230 FEI operating at 10 kV, supplied by Zeiss, Seoul, Republic of Korea). The samples had sufficient conductivity, eliminating the need for additional metal conductive coating. High-resolution transmission electron microscopy (HRTEM), dark field, and energy dispersive spectroscopy (EDS) maps were obtained using a TALOS F200X, manufactured by Thermo Fisher Scientific Korea Ltd., Seoul, Republic of Korea microscope operating at 200 kV. The crystalline structure was studied using X-ray diffraction (XRD, Rigaku D/max-2550 V, Tokyo, Japan, Cu-Ka radiation), while structural analysis was carried out using a Raman spectrum (LabRAM HR UV/vis/NIR Horiba Jobin-Yvon, Palaiseau, France) and chemical analysis using X-ray photoelectron spectroscopy (Sigma Probe Thermo VG spectrometer using Mg Kα X-ray sources, Waltham, MA, USA). The XPS spectra were curve-fitted with a mixed Gaussian–Lorentzian shape using the freeware XPSPEAK version 4.1. BET surface area and porosity were measured using Nitrogen adsorption and desorption isotherms at 77 K using the Belsorp Mini II Surface Area and Pore Size Analysis system by BEL Japan Inc. (Toyonaka, Japan). 

Electrochemical testing was carried out in a conventional three-electrode system using a Bio-Logic electrochemical workstation manufactured by Biologic, Seyssinet-Pariset, France. The working electrode was prepared by coating a catalyst ink on a glassy carbon electrode (3.0 mm in diameter) using a 5% *w*/*w* solution in water and 1-propanol as the binder to stabilize the catalyst. Prior to each electrochemical test, the glassy carbon electrode was thoroughly cleaned and polished using 1 μm and 100 nm alumina powder and followed by sonication in ultrapure water for a few seconds and natural drying. A reversible hydrogen electrode (RHE) was used as the reference electrode, and a graphite rod was used as the counter electrode. The catalyst ink for the fabrication of the working electrode involved mixing 5 mg of the catalyst, 50 μL of Nafion solution and 450 μL of ethanol in a sonicator for 30 min to form a uniform catalyst suspension. Subsequently, 20 μL of the catalyst suspension was added drop-wise to the glassy carbon electrode, naturally dried, and used for electrochemical testing as a working electrode. Cyclic voltammetry (CV) was used to evaluate the catalytic performance at various scanning rates ranging from 1 mV s^−1^ to 10 mV s^−1^ for urea oxidation, and the electrolyte was 1.0 M KOH or 1.0 M KOH/0.33 M urea. 

### 2.2. Synthesis of NMP Based Ionic Liquid

The coordination ionic liquid of C_5_H_9_NO.0.2NiCl_2_ was prepared by reacting NMP with NiCl_2_. Since the process of forming NMP-NiCl_2_ ionic liquid is exothermic, the reaction was carried out in a reactant flask immersed in an ice bath maintained at 0 °C. In this process, 0.02 mol (2.6 g) of NiCl_2_ was added to 0.1 mol (9.91 mL) of NMP over a period of 60 min under constant stirring while ensuring no abrupt heat increase. After the complete addition of NiCl_2_, the mixture was stirred for an additional 60 min at 0 °C and then allowed to come to room temperature naturally, after which liquid ionic liquids could be obtained.

### 2.3. Synthesis of Ni-MOF@rGO

A one-pot microwave procedure was used to concomitantly reduce and grow Ni-MOF nanorods anchored on graphene oxide. In this process, a 50 mL solution containing 2 mg/mL of graphene oxide dispersed in NMP was prepared. To this solution, 50 mL of C_5_H_9_NO.0.2NiCl_2_ and 1 g of waste PET were added, and the mixture was subjected to microwave irradiation for 180 s. The hot mixture was shaken vigorously, and this process was repeated until no visual PET was observed (usually three times). Subsequently, the reaction mixture was kept overnight (8 h) in an oven at 100 °C. Finally, the solvent (NMP) was removed by filtration and dried at 175 °C to eliminate any traces of NMP and obtain Ni-MOF@rGO.

## 3. Results and Discussion

**Figure 1:**
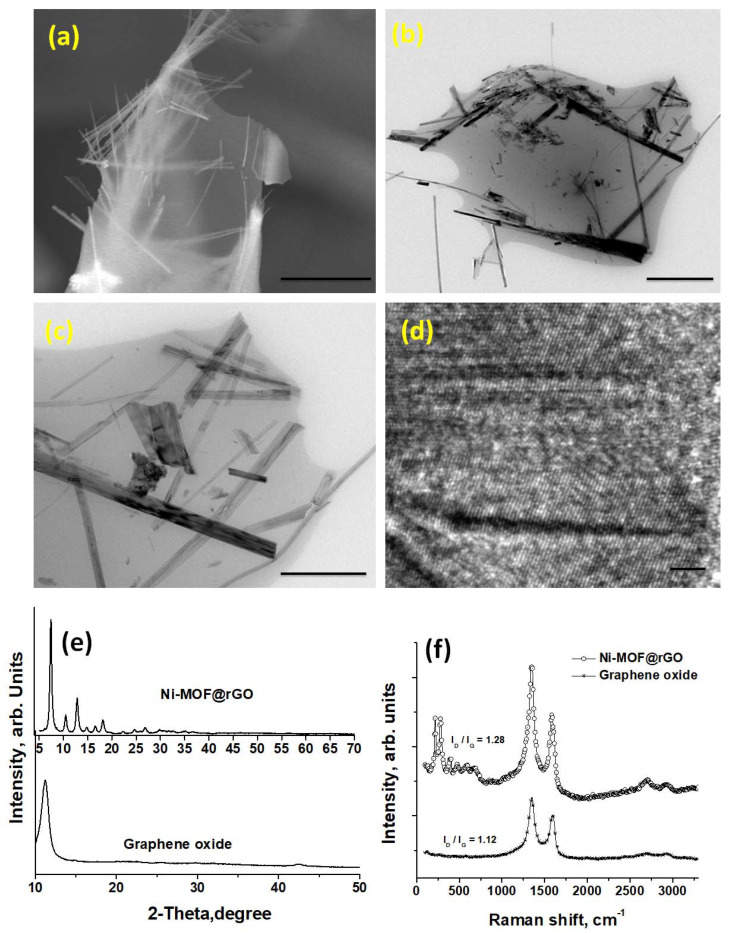
Representative SEM (**a**) and TEM at low (**b**), medium (**c**), and high (**d**) magnifications of Ni–MOF@rGO. XRD (**e**) and Raman spectra (**f**) of graphene oxide and Ni–MOF@rGO. Scale bars are 2 μm, 1 μm, 500 nm and 10 nm in Figure (**a**–**d**), respectively.

The morphology and microstructure of the Ni-MOF@CNT synthesized through the graphene oxide-mediated depolymerization of waste PET in NMP-NiCl_2_ coordination ionic liquid were studied using SEM (Figure 1a). The SEM images revealed nanorods with diameters of 150–300 nm and lengths of several micrometers, which were well dispersed on the surface of the reduced graphene oxide. The mechanism of formation of Ni-MOF can be envisaged in three steps. The formation of Ni-MOF nanorods can be described as a sequential process involving melting, dissolving, depolymerization, and MOF formation. In the first step, due to the heat generated by microwave radiation, PET will be dissolved in NMP, which will eventually form an evenly dispersed emulsion of PET chains in the NMP ionic liquid. Subsequently, the functional ester groups in the melted and dissolved PET chains will be attacked by the nickel moieties of the ionic liquid, initiating random chain scission and leading to the formation of low molecular weight oligomers. With further reaction time, these oligomers will eventually be depolymerized to form terephthalic acid (TPA) and oxalic acid (OA) as byproducts [45]. TPA will react with the nickel moieties to form Ni-MOF, while OA acts as a synthesis modulator through the k^2^ chelation mode of oxalic acid, resulting in high stability of the resulting five-membered ring [46].

Additionally, graphene oxide can also depolymerize PET. Chemically, GO can be represented as C_140_H_42_O_20_, with most of the oxygen moieties existing as hydroxyl (-OH), carboxyl (-COOH), alkoxy (-O-R), or carbonyl (-C=O) groups [47]. These oxygenated moieties participate in depolymerization through solvolytic chain cleavage, which involves breaking the ester linkage of PET. This process eventually leads to the formation of terephthalic acid monomers through a trans-esterification reaction occurring between the oxygen moieties of GO and the ester linkages in PET. One of the byproducts of PET depolymerization in air is the formation of oxalic acid, which is beneficial and is known to impart excellent crystallinity [48]. This does not affect the MOF structure and composition, which is evident from the HR-TEM image shown in Figure 1d and confirmed by the XRD spectra exhibited in Figure 1e. The XRD peak of graphene oxide is featureless except for the broad peak at 10.2 which is typical of graphene oxide [48]. The XRD of Ni–MOF@rGO shows sharp peaks at 7.45 and 12.8, corresponding to the (110) and (300) nickel peaks of Ni-MOF with terephthalic acid linkers [49]. In addition to these two peaks, minor peaks of nickel at 10.5, 18.2, 26.93, 30.14, 35.29, and the (110) peak of carbon at 24.6 can also be observed. The crystal size of the MOF, calculated using the Scherrer method, was approximately 9.06 µm, which is nearer to the reported value of 9 µm in TPA-based Ni-MOF [50].

More information on the structural changes can be obtained from Raman spectra exhibited in Figure 1f. The Raman spectra of Ni–MOF@rGO show two major nickel-associated peaks observed at approximately 214 and 280 cm^−1^ and their corresponding satellite peaks at 474 and 589 cm^−1^. These peaks can be attributed to low symmetry first-order phonon scattering arising due to lattice defects. In addition to the nickel peaks, the well-known disorder-induced Raman-active D-band peak of graphene at 1357 cm^−1^ was observed, which was attributed to the in-plane defects of graphene and the presence of MOF nanorods on its surface. The peak observed at 1578 cm^−1^ in Ni–MOF@rGO represents the G-band of sp^2^-bonded carbon, whereas this same peak was observed at 1587 cm^−1^ in graphene oxide. The ratio of D-band to G-band intensities (I_D_/I_G_ ratio) was 1.28 in Ni–MOF@rGO, which was higher than that of graphene oxide with a ratio of 1.12. The Raman spectra were also used to measure the number of layers of graphene. The calculations showed a value of 1.28 [51], indicating that Ni-MOF nanorods anchored on mono- or bi-layered reduced graphene oxide can be synthesized from our newly developed technique.

**Figure 2:**
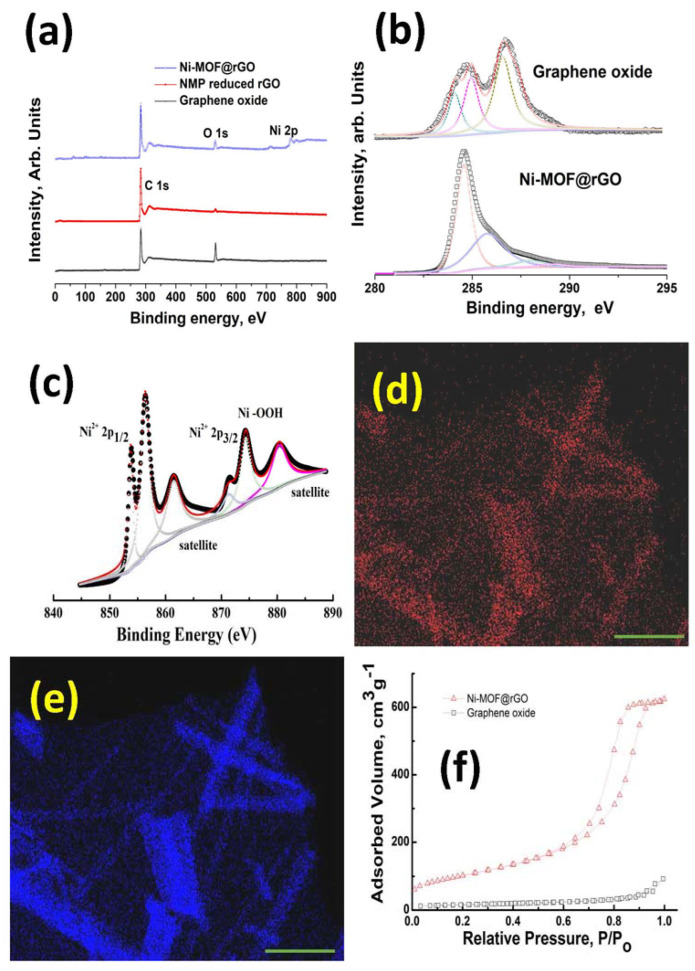
XPS survey scans of graphene oxide, NMP-reduced GO, and Ni–MOF@rGO (**a**). Deconvoluted C1 s XPS spectra of graphene oxide and Ni–MOF@rGO (**b**) and Ni 2p of Ni–MOF@rGO (**c**). HRTEM elemental maps of Ni–MOF@rGO: oxygen (**d**) and nickel maps (**e**). Surface area of graphene oxide and Ni–MOF@rGO (**f**). Scale bars in Figure (**d**,**e**) represent 500 nm.

An in-depth XPS study was carried out to investigate the changes in the chemical composition of GO, NMP-reduced graphene, and Ni–MOF@rGO. The survey scans plotted in the range of 0 to 900 eV in Figure 2a show that in GO, two sharp peaks corresponding to C 1s and O 1s peaks were observed. In case of NMP-reduced GO, the O 1s peak almost disappeared, with only C 1s peak dominating in the measured frequency range. However, in the case of Ni–MOF@rGO, a Ni 2p peak in the range of 840–890 eV was observed in addition to the C 1s and O 1s peaks. The C/O ratio, which represents the ratio of carbon to oxygen moieties, is an important parameter to quantify the extent of reduction of graphene oxide to rGO. The C/O ratio of GO, NMP-reduced graphene, and Ni–MOF@rGO calculated from the plots shown in Figure 2a increased from 1.68 in graphene oxide to 4.35 and 5.32 in NMP-reduced graphene and Ni–MOF@rGO, respectively. The ability of NMP to reduce oxidized carbonaceous solids has been attributed to the cleavage of carbon–oxygen bonds by the pyrrolidinone moiety at high temperatures.

XPS spectroscopy is a powerful tool for studying the electronic structure of nickel moieties. The XPS spectra of the Ni 2p binding energy region ranging from 840–890 eV plotted in Figure 2c shows typical nickel 2p doublet peaks centered at 855.8 and 873.8 eV, corresponding to Ni 2p^3/2^ and Ni 2p^1/2^, respectively. Two satellite peaks centered at 860.76 and 879.8 eV were also observed. The peak at 855.8 eV is normally assigned to OH-Ni-OH bonds [52], which in our case proves that our newly developed graphene oxide-mediated depolymerization of waste PET in NMP-NiCl_2_ ionic liquid results in the formation of Ni-MOF nanorods. The peaks at 873.8 and 860.8 eV were assigned to Ni^2+^ [49]. The observed satellite peak at 879.8 provides further evidence that nickel is in the valence state of Ni^2+^ in our Ni-MOF nanorods. This was further confirmed by HRTEM EDS maps, which showed that nickel (Figure 2d) and oxygen (Figure 2e) moieties existed concomitantly. Figure 2f shows the nitrogen adsorption isotherms measured at 77 K for GO and Ni–MOF@rGO. The Ni–MOF@rGO isotherm showed a typical type II adsorption isotherm, indicating the formation of adsorbate multilayers. The corresponding BET surface area showed tremendous increase from 45 m^2^/g in GO to 641 m^2^/g in Ni–MOF@rGO, which can be attributed to the Ni-MOF anchored on the rGO surface acting as spacers, thereby inhibiting the restacking of graphene through van der Waals forces.

**Figure 3:**
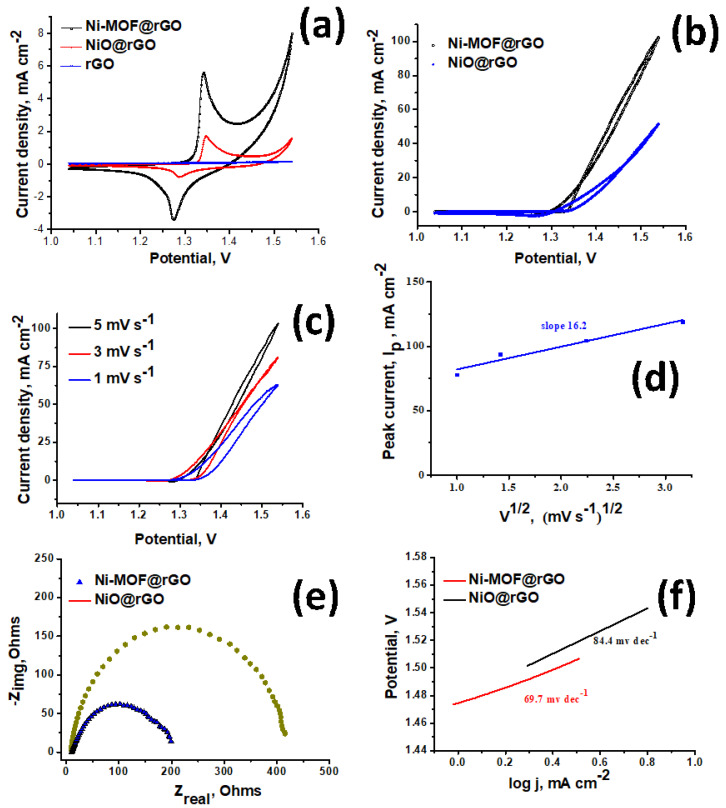
CV curves for Ni–MOF@rGO, NiO@rGO, and rGO in 1.0 M KOH solution (**a**); urea electro-oxidation LSV curves for Ni–MOF@rGO and NiO@rGO in 1.0 M KOH solution (**b**); effect of scan rate on LSV behavior in Ni–MOF@rGO (**c**) and its corresponding peak current analysis (**d**); Nyquist curves (**e**) and Tafel plots (**f**) for Ni–MOF@rGO and NiO@rGO in 1.0 M KOH with 0.33 M urea.

The applicability of our newly developed Ni–MOF@rGO as electro-catalyst in urea-enhanced water splitting was investigated. The suitability of Ni–MOF@rGO, NiO@rGO, and rGO electrodes was first evaluated for their water oxidation ability in a standard 1.0 M KOH solution. In the case of rGO, no visually discernible peak was observed, indicating that rGO alone is incapable of water splitting. Among the two nickel electro-catalysts tested, Ni–MOF@rGO showed a substantially better water splitting ability compared to NiO@rGO. This can be attributed to the unique electronic structures of nickel in Ni–MOF@rGO, where the nickel cations are bound by terepthalic acid ligands in OH-Ni-OH (as evidenced from XPS data). This configuration is known to be more electroactive compared to the electronic state of nickel in NiO@rGO, where the nickel particles in the oxidized state show lower conductivity and decreased electrocatalytic activity [53]. After confirming the utility of both nickel-based electrodes in water oxidation, we shifted our attention to studying the electrocatalytic activity of Ni–MOF@rGO and NiO@rGO in the urea oxidation reaction (UOR) in an alkaline aqueous electrolyte consisting of 0.33 M urea in 1.0 M KOH. The LSV curves of Ni–MOF@rGO and NiO@rGO plotted in Figure 3b show that the highest urea oxidation current observed in Ni–MOF@rGO electrocatalysts was more than double that of NiO@rGO. This can be attributed to the unique electronic structure of nickel moieties in the OH-Ni-OH configuration, which deprotonates a surface hydroxide group, forming the electroactive Ni^3+^ species that then oxidizes urea [54]. Additionally, the onset potential of Ni–MOF@rGO for urea oxidation was also substantially lower than that of NiO@rGO, implying that our newly developed Ni–MOF@rGO was a more energy-efficient electrocatalyst for urea oxidation reactions compared to traditional nickel oxide-based electrocatalysts. The electrokinetic studies of urea electrooxidation by the Ni–MOF@rGO electrocatalyst was conducted by varying the scan rate (Figure 3c). With an increasing scan rate, a linear increase in current density (Figure 3d) was observed, indicating the dominance of a diffusion-controlled process. This observation was further confirmed by the Nyquist curves and Tafel plots exhibited in Figure 3e, and 3f, respectively. The substantially lower diameter of Ni–MOF@rGO compared to NiO@rGO in the Nyquist plot indicates an improved charge transfer rate and rapid kinetics during the urea oxidation process. The Tafel slope of Ni–MOF@rGO (69.7 mV dec^−1^) was substantially lower than that of NiO@CNT (84.4 mV dec^−1^). The urea electrooxidation process is a very complex phenomenon in which a six-electron process occurs, and there is some controversy regarding the exact mechanism. However, the general consensus is that during the UOR reaction, electrochemical oxidation of Ni^3+^ and Ni^2+^ of the electrocatalysts to nickel oxyhydroxides occurs. XPS studies, as discussed above, have shown that nickel moieties are in OH-Ni-OH state in Ni–MOF@rGO, which is electrochemically more active than the tightly bound oxides in NiO@rGO, thereby exhibiting better electrocatalytic activity [55].

**Figure 4:**
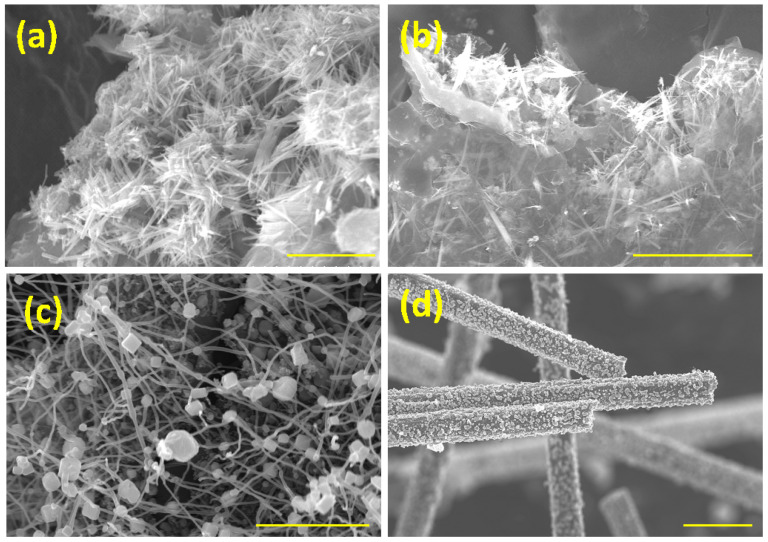
Representative morphology of Mn-MOF (**a**) and Fe-MOF (**b**) nanorods derived from NMP-MnCl_2_ and NMP-FeCl_3_, respectively; Ni-MOF nanocubes anchored on CNT (**c**) and Ni-MOF nano-islands on carbon fiber (**d**). Scale bars are 3μm, 2μm, 500 nm, and 3μm in Figure (**a**–**d**), respectively.

In order to demonstrate the versatility of our newly developed method for transforming waste PET into MOF nanostructures using NMP-based ionic liquid, we investigated the effects of other coordination ionic liquids such as NMP-MnCl_2_ and NMP-FeCl_3_ in the presence of graphene oxide. Representative SEM micrographs of Mn-MOF (Figure 4a) and Fe-MOF (Figure 4b) show micrometer-long, nanometer-thick Fe and Mn-MOF nanorods anchored on rGO substrates, which is consistent with the data observed with nickel. However, when the substrate was changed to CNT, no nanorods were formed, but nanometric cube-shaped nickel MOF-CNT structures were formed (Figure 4c). 

A possible explanation for this can be attributed to the nanoscale curvature effect of the CNT surface, which results in minimal contact between the MOF and CNT surface, thereby inhibiting the growth of one-dimensional nanostructures. Changing the substrate to carbon fiber resulted in the formation of MOF “nano-islands” (Figure 4d). This variation in the shape of MOFs on carbon fiber can be attributed to the growth kinetics. The typical growth of one-dimensional nano structures such as nanorods or nanobelts involves two distinct but concurrent steps: homogeneous nucleation and spontaneous agglomeration. In the case of carbon fiber substrates, spontaneous agglomeration appears to dominate when compared to nucleation. Despite the differences in morphology, these results prove that nanostructured MOFs can be grown on a wide range of substrates using our newly developed NMP-based IL depolymerization technique. These results prove that waste PET can be transformed into MOFs of various shapes anchored on carbonaceous substrates through our newly developed coordination ionic liquid-induced depolymerization.

## 4. Conclusions

In summary we have reported transformation of waste PET into Ni-MOF nanorods in an NMP-based coordination ionic liquid. Morphological studies by SEM and TEM revealed mesoporous 3D nanostructures with nanometer-thick and micrometer-long Ni-MOF nanorods anchored on rGO. Structural analysis using XRD showed highly crystalline Ni-MOF nanorods, while Raman spectra showed bilayer-reduced graphene oxide. High-resolution chemical analysis using XPS showed that nickel moieties exist in an electroactive OH-Ni-OH state. When applied as electrocatalysts in the urea oxidation reaction, our newly developed Ni–MOF@rGO electrodes exhibited a lower onset potential, increased current responses, faster kinetics of urea oxidation, and lower charge transfer resistance. This enhanced UOR performance was attributed to the mesoporous structure of well-dispersed electroactive Ni-MOF nanorods distributed on conductive rGO substrates. The applicability of our newly developed depolymerization technique in synthesizing various MOF nanostructures such as cubes and particles on carbon nanotubes and carbon fibers was also demonstrated.

## Data Availability

The data presented in this study are available from the corresponding author upon request.
